# Citrus Disease Detection Based on Dilated Reparam Feature Enhancement and Shared Parameter Head

**DOI:** 10.3390/s25071971

**Published:** 2025-03-21

**Authors:** Xu Guo, Xingmeng Wang, Wenhao Zhu, Simon X. Yang, Lepeng Song, Ping Li, Qinzheng Li

**Affiliations:** 1 School of Big Data and Automation, Chongqing Chemical Industry Vocational College, Chongqing 401228, China; guoxu@cqcivc.edu.cn; 2 School of Electronic and Electrical Engineering, Chongqing University of Science & Technology, Chongqing 401331, China; 2022204009@cqust.edu.cn (X.W.); 2022204029@cqust.edu.cn (W.Z.); 2022441115@cqust.edu.cn (Q.L.); 3 Advanced Robotics and Intelligent Systems Laboratory, School of Engineering, University of Guelph, Guelph, ON N1G 2W1, Canada; syang@uoguelph.ca; 4 Chongqing Academy of Agricultural Sciences, Chongqing 400039, China; lipinghanxe2005@126.com

**Keywords:** YOLOv8n-DE, dilated reparam feature enhancement, shared parameter head, citrus disease detection, real-time detection

## Abstract

Accurate citrus disease identification is essential for targeted orchard pesticide application. Current models struggle with accuracy and efficiency due to diverse leaf lesion patterns and complex orchard environments. This study presents YOLOv8n-DE, an improved lightweight YOLOv8-based model for enhanced citrus disease detection. It introduces the DR module structure for effective feature enhancement and the Detect_Shared architecture for parameter efficiency. Evaluated on public and orchard-collected datasets, YOLOv8n-DE achieves 97.6% classification accuracy, 91.8% recall, and 97.3% mAP, with a 90.4% mAP for challenging diseases. Compared to the original YOLOv8, it reduces parameters by 48.17%, computational load by 59.26%, and model size by 41.94%, while significantly decreasing classification and regression errors, and false positives/negatives. YOLOv8n-DE offers outstanding performance and lightweight advantages for citrus disease detection, supporting precision agriculture development in orchards.

## 1. Introduction

Disease prevention and control play a critical role in crop growth by mitigating economic losses and enhancing crop quality [1]. Citrus plants are no exception, as they face various disease threats. Accurate and timely identification and management of these diseases are essential to ensure environmentally sustainable citrus production [2].

Common citrus diseases, such as Huanglongbing (HLB) [3], anthracnose, and melanose, are notoriously challenging to detect due to the variability in lesion shapes, complex orchard environments, disease interactions, and the dynamic angles of leaf growth [4]. The introduction of deep learning convolutional neural networks (CNNs) [5] in 2017 has significantly improved the generality and accuracy of pest and disease identification. Kurmi et al. [6] proposed a generalized deep convolutional neural network-based model for leaf disease detection, achieving 95.35% accuracy on the PlantVillage dataset. Accurate identification of citrus diseases is critical for precision pesticide management, yet existing detection models struggle to balance accuracy, computational efficiency, and adaptability to complex orchard environments. While recent studies have achieved notable progress—including CNN-based approaches achieving 95–98% accuracy in controlled settings [7,8,9,10,11] and enhanced YOLO variants attaining 93.2% AP for fruit detection [12]—three critical limitations persist. First, most methods rely on small-scale datasets (e.g., 751 images [7]) or simplified environments, limiting their robustness against diverse lesion patterns and field conditions. Second, the computational complexity of hybrid architectures like TRL-GAN [13] and attention networks [11] hinders deployment on edge devices. Third, existing models exhibit significant performance degradation when detecting nutrition deficiencies and morphologically similar diseases (e.g., Huanglongbing vs. mosaic yellowing [13]), with reported false negative rates exceeding 16% in multi-class scenarios [10,14]. Current studies fail to address the dual requirements of keeping the model lightweight while also attaining high accuracy in citrus disease recognition, which constitute essential prerequisites for scalable precision agriculture implementations.

Despite these advancements [15], detecting citrus diseases under diverse environmental conditions remains challenging. Variations in scale, illumination, and background necessitate models capable of addressing these complexities. Balancing detection speed and accuracy requires optimizing model complexity and inference efficiency. Therefore, those developing and testing citrus disease detection models must aim to enhance their robustness and accuracy across varying viewpoints [16], backgrounds, and lighting conditions [17,18].

This study focuses on detecting diseases in citrus plants, particularly key leaf diseases such as Huanglongbing [19], citrus canker, anthracnose, and melanose. The primary goal is to improve detection accuracy and speed, supporting orchard management and precision agriculture. The innovative contributions of this paper are outlined below:

1. Proposing the DR module, which enhances multi-scale feature extraction through dilated convolutions and re-parameterization techniques.
2. Designing the Detect_Shared detection head to reduce redundant parameters by leveraging partial convolution and channel fusion.
3. Achieving a balanced design that integrates lightweight performance with high accuracy.

The remainder of this paper is organized as follows. Section 2 describes the proposed methodology and network architecture in detail. Section 3 outlines the experimental setup, including dataset composition, training environment, and evaluation metrics. Section 4 presents the analysis of experimental results from five perspectives. Section 5 provides a model visualization analysis. Finally, the paper concludes with a summary and discussion of the author’s contributions.

## 2. Materials and Methods

**Figure 1:**
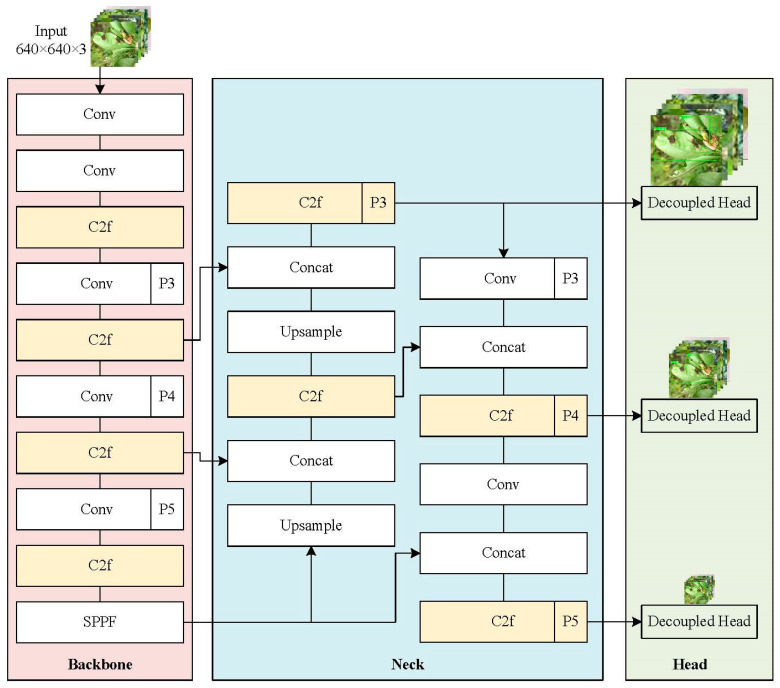
YOLOv8 network architecture. “Conv” represents convolution; “C2f” denotes the critical feature extraction component; “SPPF” denotes the Spatial Pyramid Pooling module; “Concat” indicates feature fusion; “Upsample” represents upsampling; and “Decoupled Head” represents the detection head structure separating classification and regression tasks.

**Figure 2:**
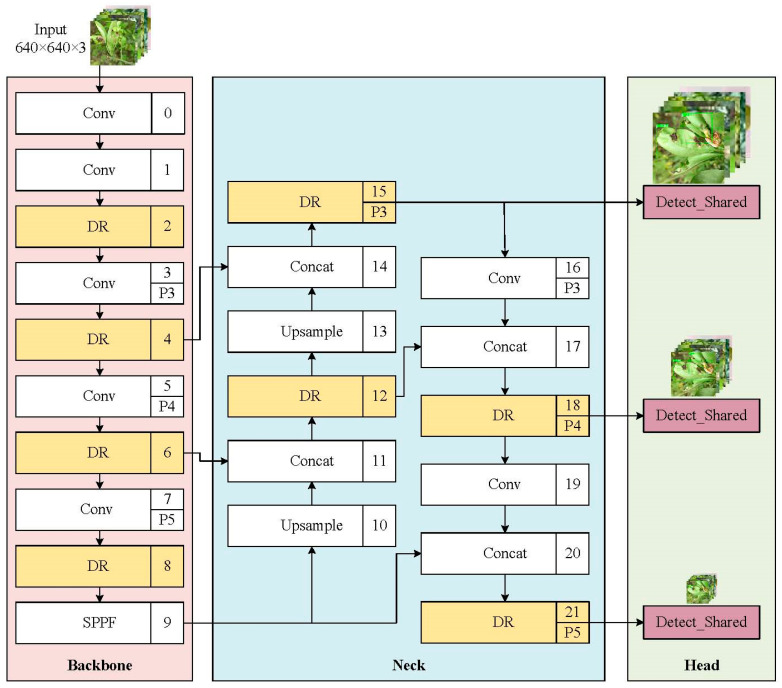
YOLOv8n-DE citrus disease detection model. “DR” refers to the Dilated Reparam Feature Enhancement module, and “Detect_Shared” refers to the Shared Parameter Head.

This paper proposes a fast citrus disease detection model based on Dilated Reparam Feature Enhancement (DR module) and a shared parameter head named the YOLOv8n-DE citrus disease detection model. The model uses the YOLOv8 object detection algorithm as the baseline model [20], with modifications enabling it to swiftly and accurately identify citrus diseases in orchard environments. This paper introduces an innovative and efficient feature extraction-fusion module with reduced parameters and computational demands, referred to as the DR module. Enhancing feature representation, the method accurately captures and highlights the critical features required for object detection tasks. Additionally, to address the issue of high parameter counts in the YOLOv8 coupled head, this paper designs an efficient shared parameter head to reduce redundant computations and memory access. This shared parameter head structure performs unified convolution first, followed by separate classification and regression tasks, allowing the model to maintain high disease detection accuracy while reducing the number of parameters. The YOLOv8 network architecture is illustrated in Figure 1, while Figure 2 presents the architecture of our proposed YOLOv8n-DE citrus disease detection model. Building upon the backbone, neck, and head components of YOLOv8, the YOLOv8n-DE model introduces structural enhancements: (1) the Dilated Reparametrization (DR) module incorporated in the backbone and neck improves feature extraction capabilities; and (2) a parameter-shared detection head (Detect_Shared) optimizes detection speed and computational efficiency.

### 2.1. DR Module

**Figure 3:**
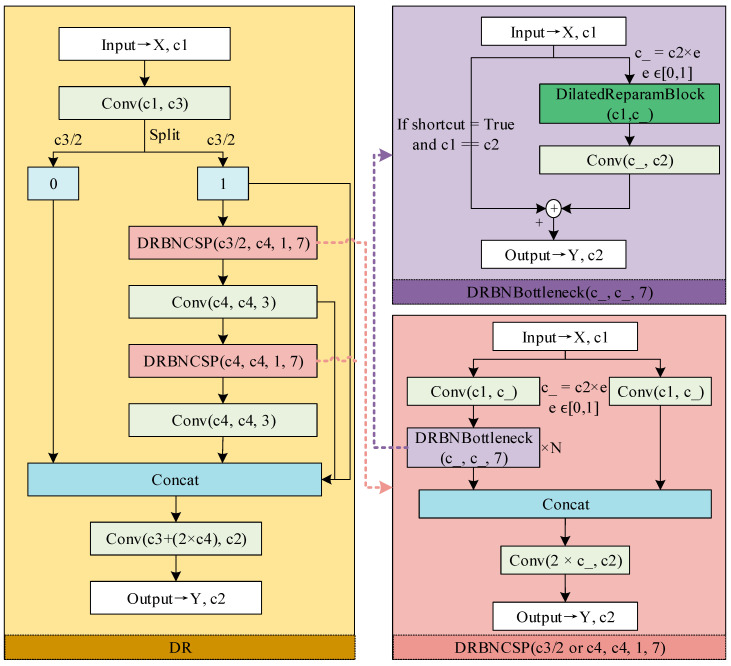
The structure of the DR module.

The YOLOv8n model employs the C2f (Faster Implementation of CSP Bottleneck with 2 convolutions) module as a core component to enhance model performance and accuracy. Its residual connections and bottleneck design enable the model to handle complex image features effectively while reducing overall complexity. In the citrus leaf disease extraction task, the DR module designed in this paper achieves superior feature extraction compared to the C2f module. It significantly improves disease detection accuracy while effectively reducing the number of parameters. The structure of the DR module is shown in Figure 3. The DR module employs dilated convolutions to capture multi-scale disease characteristics, including the punctate lesions of Anthracnose, elevated canker lesions, yellowing patterns in Huanglongbing, melanose induced black mold spots, ambiguous and diverse nutritional deficiency symptoms, and leaf perforations caused by Podagricomela nigricollis. Through parameter reparametrization, this module reduces redundant small convolution stacking, enabling enhanced focus on holistic disease features rather than localized artefacts. Notably, the dilated convolution and reparametrization techniques are specifically optimized for citrus disease classification rather than generic image recognition tasks.

By introducing the Dilated Reparam Block, a DRBNBottleneck structure was designed based on this module, leading to the development of a DRBNCSP. The DRBNCSP module optimizes the traditional NCSPELAN module, ultimately achieving the design of DR module. This section further explains the DR module, the DRBNCSP submodule, the DRBNBottleneck submodule, and the Dilated Reparam Block.

The DR module, depicted in Figure 3, first divides the input feature map into two equal parts along the channel dimension, each containing c_3_/2 channels. This design enhances the model’s nonlinear expression capabilities. Instead of directly adopting a bottleneck design, the module introduces two DRBNCSP submodules for channel transformation. Following the DRBNCSP submodules, 3 × 3 convolutional layers are added to adjust the number of feature map channels and further process and integrate the features. By embedding the DRBNBottleneck module within the DRBNCSP, the design achieves multi-level and multi-scale feature extraction and fusion. This approach enhances multi-scale information transmission and integration while reducing the risk of overfitting.

**Figure 4:**
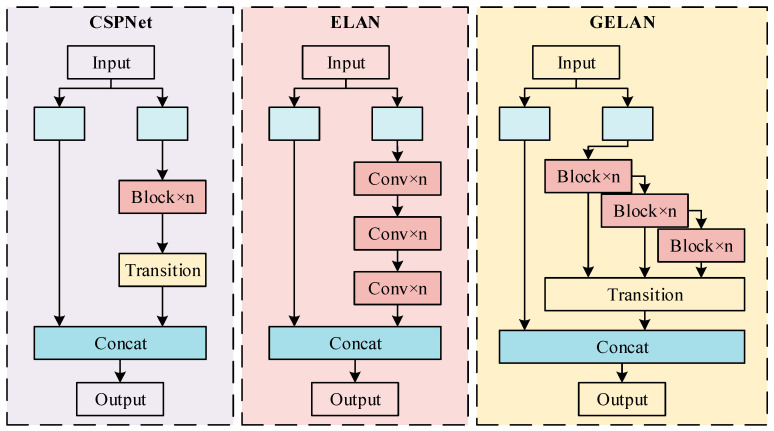
Different aggregation networks.

The DRBNCSP submodule, as illustrated in Figure 3, combines the Generalized Efficient Layer Aggregation Network (GELAN) concept. GELAN, introduced in the YOLOv9 algorithm [21], integrates CSPNet with Gradient Path Planning and ELAN neural network structures, balancing lightweight design, inference speed, and accuracy (Figure 4). While the RepNCSP module in YOLOv9 adopts the RepNBottleneck module for reparameterization, this paper presents a DRBNBottleneck. The DRBNBottleneck demonstrates significant advantages in expanding the receptive field and capturing complex spatial information. It is particularly suited for disease identification tasks requiring the capture of long-range dependencies and features in complex orchard environments.

Traditional convolutional network architectures often increase the receptive field, improve abstraction levels, and enhance general representation capacity by adding small convolutional kernels. However, stacking many small kernels leads to significant parameter and computational overhead. Furthermore, the marginal returns from stacking small kernels diminish under model size constraints. This paper adopts large-kernel convolutions, which deliver state-of-the-art performance and are comparable to transformers [22] for feature extraction. Specifically, we use a limited number of large convolutional kernels to ensure a broad receptive field, combine small convolutions to enhance feature abstraction levels, and employ an efficient reparameterization structure to deepen the model, thereby improving its general representational capacity [23].

The DRBNBottleneck submodule, illustrated in Figure 3, employs a Dilated Reparam Block structure. The model gains significant advantages in feature extraction capability, receptive field expansion, and overall efficiency by integrating dilated convolutions with reparameterization techniques [24]. Large convolutional kernels are deliberately designed to accommodate the varying spatiotemporal receptive fields required for distinguishing multiple citrus disease types.

In the Dilated Reparam Block, the original convolution kernel is represented as:

(1) $$W\in {ℜ}^{k\times k}$$

where *W* represents the original convolution kernel (filter); *k* × *k* indicates it is a matrix of dimension *k* × *k*; and ℜ denotes the real matrix.

In dilated convolution, inserting zeros into the convolution kernel increases its effective size:

(2) $$W^{\prime }\in {ℜ}^{(\left(k-1\right)r+1)\times \left(\left(k-1\right)r+1\right)}$$

where $W^{\prime }$ represents the dilated convolution kernel; *r* is the dilation factor; and *k* is the size of the dilated convolution kernel.

Thus, a dilated convolution layer with a small kernel can be equivalently transformed into a non-dilated layer with a larger sparse kernel. This transformation is implemented via transposed convolution with stride:

(3) $$W^{\prime }=conv\_transpose2d\left(W,I,stride=r\right)$$

where *I* is the unit convolution kernel.

**Figure 5:**
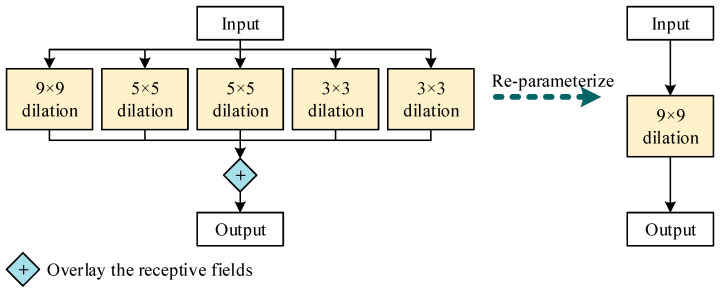
Dilated Reparam Block.

As shown in Figure 5, the Dilated Reparam Block simulates a large receptive field by stacking convolution kernels with different dilation rates. The output feature maps of these kernels are merged along the channel dimension to achieve a large receptive field effect. This approach avoids the high computational cost of directly using large convolution kernels while effectively expanding the receptive field and preserving contextual information.

Small convolution kernels with varying dilation rates can be reparameterized into a single 9 × 9 large receptive field convolution layer. This technique ensures computational efficiency while maintaining the model’s ability to capture long-range dependencies and contextual information.

### 2.2. Efficient Head Design

The detection head in object detection models is responsible for handling classification and regression tasks [25]. YOLO series detection algorithms typically feature two types of detection heads: coupled heads and decoupled heads. For example, YOLOv5 uses a coupled head design, while YOLOv8 employs a decoupled head design. This paper investigates detection heads and proposes a shared parameter head that is more suitable for disease detection tasks.

#### 2.2.1. Coupled Head

**Figure 6:**
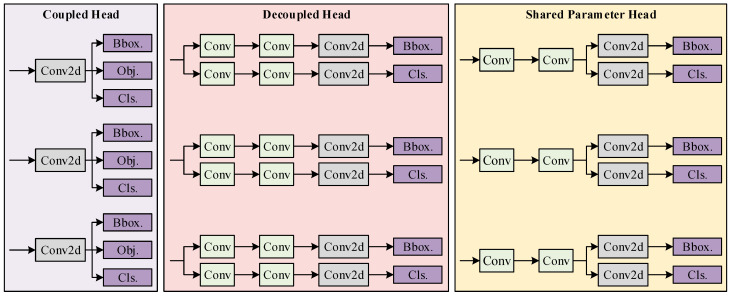
Comparison of coupled head (YOLOv5), decoupled head (YOLOv8), and shared parameter head structure.

Figure 6 shows the structure of the coupled head. In a coupled head, classification and regression tasks share a common set of parameters, with outputs generated directly through convolution. Task separation occurs only during the final loss computation. This design reduces the number of parameters and computations while enhancing detection speed. However, the convolutional layers in the coupled head are simultaneously tasked with feature extraction for classification and regression. This shared parameter design weakens the feature extraction capability, leading to suboptimal performance for both tasks.

#### 2.2.2. Decoupled Head

The structure of the decoupled head is also shown in Figure 6. In a decoupled head, classification and regression each have a separate branch, allowing independent feature extraction. The clear task division in the decoupled head structure enables each branch to capture more task-specific features, improving detection accuracy. However, the separate branches for each task double the number of parameters and computations, significantly increasing inference time. In the baseline YOLOv8 model, the decoupled head accounts for 3.64 GFLOPs (Giga Floating Point Operations per Second, a unit of computation), constituting 45% of the model’s total computation (8.1 GFLOPs).

#### 2.2.3. Shared Parameter Head

**Figure 7:**
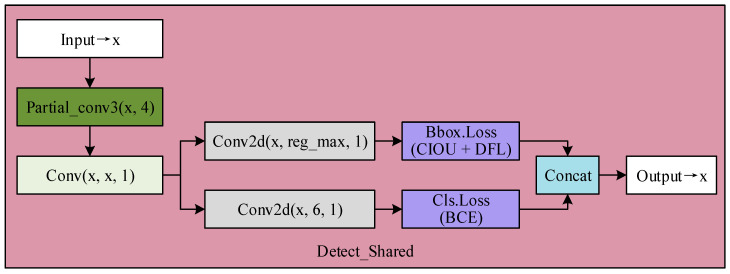
Structure of the shared parameter head.

To address the shortcomings of the coupled head in disease detection and the excessive parameters and computation of the decoupled head, this paper combines both designs to propose a shared parameter head. The proposed efficient detection head, Detect_Shared, is illustrated in Figure 7.

We merge computations for the input feature map list and then apply separate convolutions to reduce the parameters, achieving a lightweight design. Initially, the Partial_conv3 module performs partial convolution operations on the input tensor [26]. Only a subset of channels is convolved in partial convolution, while the other channels remain unchanged. This method effectively handles sparse data, preventing the computational overhead associated with full convolution. Partial convolution operations prove particularly effective for handling the sparse lesion distributions characteristic of citrus disease imagery. After the partial convolution, a 1 × 1 convolution is applied for channel fusion and dimensionality reduction. To retain the flexibility and independence of the original decoupled head, the detection head is split into two branches for convolution, where classification and regression are computed separately. This design results in the new shared parameter head, as shown in Figure 7.

The shared parameter head enhances the handling of sparse data and improves computational efficiency, providing more robust feature extraction capabilities and greater flexibility. By applying this head to disease detection models, we achieve three different-scale feature map outputs, effectively improving disease detection accuracy while reducing the number of parameters in the detection head.

### 2.3. Loss Function

The ultimate goal of citrus disease detection is to improve the accuracy of bounding boxes and disease labels in predicted images, thus enhancing classification and regression precision [27]. A loss function is used to measure the discrepancy between predicted outputs and actual annotation labels to train a model for citrus disease detection. The loss function integrates the following components to balance object detection performance: disease target bounding box localization accuracy (Box Loss), fine-grained prediction of bounding box coordinates (*DFL* Loss, Distribution Focal Loss), and classification accuracy of disease categories (Cls Loss). As shown in Figure 7, since both Box Loss and *DFL* Loss primarily measure bounding box localization accuracy, they are output through the same branch and collectively referred to as regression loss. Cls Loss is output through another branch and is called classification loss.

The Box Loss function uses Complete Intersection over Union (*CIoU*) for calculation, ensuring that both large and small targets are accurately predicted. *CIoU* integrates multiple criteria, such as *IoU*, center point distance, and aspect ratio consistency, offering a more comprehensive and precise bounding box matching metric than standard *IoU*. Due to the diverse size and shape of citrus diseases, *CIoU* significantly enhances model performance, ensuring accurate and reliable detection of diseased leaf locations. The calculation method is as follows:

(4) $$CIoU=1-IoU+\frac{\rho^{2}\left(b,b^{g}\right)}{c^{2}}+\alpha v$$

where *IoU* represents the Intersection over Union between the predicted and ground truth bounding boxes; *ρ*(*b*, *b^g^*) is the Euclidean distance between the center points of the predicted and ground truth boxes; *c* is the diagonal length of the smallest enclosing rectangle containing both predicted and ground truth boxes; *α* is the weight factor for aspect ratio consistency; and v is the aspect ratio consistency metric.

*α* is defined as:

(5) $$\alpha =\frac{v}{\left(1-IoU\right)+v}$$

v is defined as:

(6) $$v=\frac{4}{\pi^{2}}{\left(\arctan\left(\frac{w^{g}}{h^{g}}\right)-\arctan\left(\frac{w}{h}\right)\right)}^{2}$$

where *w* and *h* are the width and height of the predicted bounding box, and *w^g^* and *h^g^* are the width and height of the ground truth bounding box.

Distribution Focal Loss (*DFL*) addresses challenges such as complex orchard backgrounds and class imbalance in disease detection tasks. It enables the network to focus on the precise location and shape of bounding boxes. The *DFL* calculation is as follows:

(7) $$DFL\left(S_{i},S_{i+1}\right)=-\left(\left(y_{i+1}-y\right)\log\left(S_{i}\right)+\left(y-y_{i}\right)\log\left(S_{i+1}\right)\right)$$

where *S_i_* is the network’s output prediction, *S_i_*_+1_ is the adjacent predicted value, *y* represents the disease label, *y_i_* is the integral value of the label, and *y_i_*_+1_ is the nearest tag integral value.

Classification loss is computed using Binary Cross Entropy with Logits Loss (BCEWithLogitsLoss), which treats each disease category as an independent binary classification problem for separate optimization. This function effectively handles situations where each citrus leaf is affected by multiple diseases, which is common in citrus disease detection tasks. BCEWithLogitsLoss mitigates gradient explosion and vanishing issues, facilitating faster convergence to optimal model parameters. The calculation method is as follows:

(8) $$ClsLoss=BCEWithLogitsLoss\left(x,y\right)$$

(9) $$BCEWithLogitsLoss\left(x,y\right)=-\frac{1}{N}\sum_{i=1}^{N}\sum_{c=1}^{C}\left[y_{i,c}\log\left(\sigma \left(x_{i,c}\right)\right)+\left(1-y_{i,c}\right)\log\left(1-\sigma \left(x_{i,c}\right)\right)\right]$$

where *N* is the number of disease samples; *C* is the number of disease categories; *x_i_*_,*c*_ is the output for the *i*-th sample in the *c*-th category (before Sigmoid activation); *y_i_*_,*c*_ is the ground truth label for the *i*-th sample in the *c*-th category (either 0 or 1); log refers to the natural logarithm; and σ(*x_i_*_,*c*_) is the Sigmoid function applied to *x_i_*_,*c*_, $\sigma \left(x_{i,c}\right)=\frac{1}{1+e^{-x_{i,c}}}$.

This paper combines Box Loss, *DFL* Loss, and *Cls* Loss to calculate the total loss function for disease image detection, as follows:

(10) TotalLoss=box_weight×CIoU+dfl_weight×DFL+cls_weight×CLSLoss

where *box_weight* is the weight parameter for Box Loss, set to 7.5 in this paper; *dfl_weight* is the weight parameter for *DFL* Loss, set to 1.5; and *cls_weight* is the weight parameter for *CLSLoss*, set to 0.5. This weight distribution helps balance bounding box precision, class prediction accuracy, and attention to hard-to-classify diseases, thereby improving the overall performance of citrus disease detection.

## 3. Experimental Setup

### 3.1. Data Acquisition

**Figure 8:**
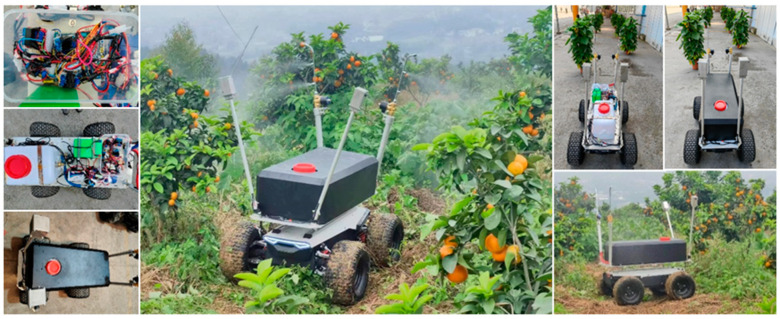
Orchard disease detection device used for disease data collection.

Existing public datasets for citrus leaves predominantly contain images of Huanglongbing, with limited data for other diseases. Field photography methods were employed to expand the dataset for additional diseases to address this gap. Citrus disease samples were collected in April 2023 from the Xianglu Mountain Citrus Orchard in Shapingba District, Chongqing (106.338928° E, 29.581242° N). Two methods were used to collect and capture citrus disease images. Method 1 involved using a Xiaomi 13 smartphone camera (Xiaomi Corporation, Beijing, China) to capture images of diseased leaves, with a camera resolution of 54 megapixels and a 12 GB + 512 GB storage capacity. The image resolutions captured were 1888 × 4096 and 3072 × 4096. Method 2 employed a camera mounted on an orchard disease detection and pesticide application device to capture real-time video of citrus trees [28]. Disease leaf images were extracted by taking three frames per second from the video. The camera on the orchard disease detection device was 5 megapixels, and the image resolution was 256 × 256. The orchard disease detection and pesticide application device is shown in Figure 8.

**Figure 9:**
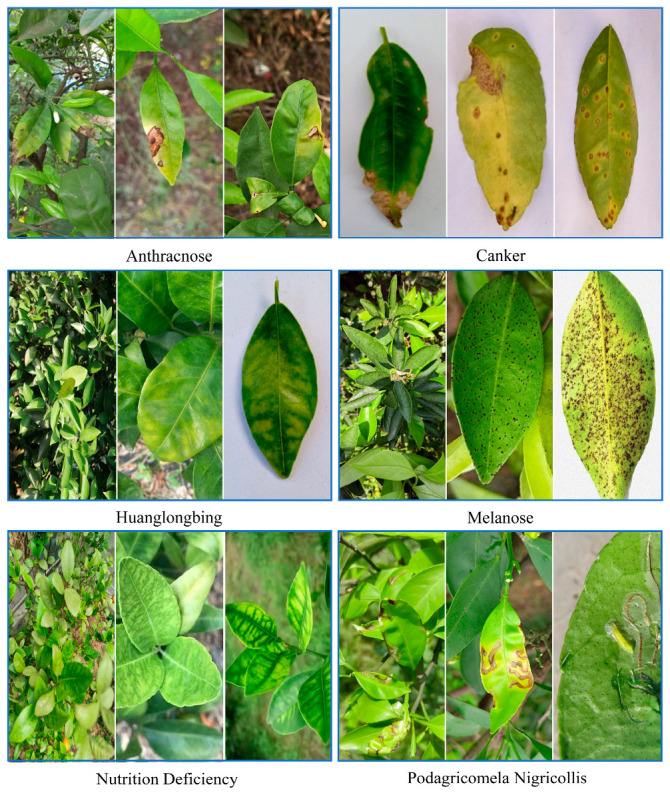
Types of citrus leaf diseases.

The survey revealed that the Xianglu Mountain Village citrus orchard primarily faces three types of diseases: anthracnose, melanose, and Podagricomela nigricollis (citrus leaf miner). To ensure the disease detection model is adaptable to various citrus orchards and to maintain the diversity of disease data and robustness of the model, additional citrus disease images were collected from four public datasets: PlantVillage [29], CCL’20 [30], Citrus Plant Dataset [31], and AI Challenger [32]. A secondary screening was conducted because some public datasets had suboptimal quality. Ultimately, images from four citrus disease datasets—Huanglongbing, Anthracnose, Canker, and Nutrition Deficiency—were selected for training and evaluating the YOLOv8n-DE model’s performance in citrus disease detection [33]. Sample disease data images are shown in Figure 9.

The LabelImg annotation software (version 1.8.6) was used to label and filter the field-collected disease data and the selected public datasets. Annotations were saved in YOLO format, including disease categories and bounding box coordinates. During the annotation process, the following category labels were defined: “a” for anthracnose, “c” for canker, “h” for Huanglongbing, “m” for melanose, “n” for nutrition deficiency, and “p” for Podagricomela nigricollis. A total of 6050 annotated citrus disease images were divided into three subsets—training, validation, and testing—at an 8:1:1 ratio. The training set contained 4840 images, the validation set contained 607 images, and the test set contained 603 images. Detailed information on the dataset is provided in Table 1.

Notably, canker disease images were not present in the field-collected data, and there were very few such images in the public datasets. However, canker is of high research value as a widespread disease of citrus trees. In this paper, only 160 sheets of canker disease were ultimately collected from publicly available datasets on the web. This imbalance in sample distribution undoubtedly increases the difficulty of the disease recognition model in identifying this category. Also, maintaining underrepresented classes provides valuable insights for detecting rare disease occurrences.

### 3.2. Experimental Environment

The citrus disease detection algorithm was trained and tested on a Linux server. The hyperparameters played a key role in the training and performance of the YOLOv8n-DE model. The experimental parameters are presented in Table 2.

During model training and testing, we implemented dynamic image scaling with input size adjustment. For original images of varying dimensions, we first calculated scaling ratios relative to the target 640 × 640 resolution. Images were proportionally resized to approximate this dimension before padding to achieve final 640 × 640 inputs, ensuring consistent model processing.

### 3.3. Evaluation Metrics

To comprehensively evaluate the training and testing performance of the disease detection model, this study employs various evaluation metrics across multiple scales to assess the model’s overall performance and detection accuracy. The complexity of the disease detection model is represented by metrics such as model size, number of parameters, and computation (GFLOPs). At the same time, the frame rate (Frames Per Second, *FPS*) is used to reflect the model’s real-time detection performance. The *FPS* calculation for the YOLOv8n-DE citrus disease detection model is as follows:

(11) $$FPS=\frac{1}{i_{t}}$$

where *i_t_* denotes the inference time per frame, and *FPS* is the reciprocal of that time, measured in frames per second (f/s), representing the number of frames the YOLOv8n-DE model can process per second.

This study uses COCO evaluation metrics—Precision (*P*), Recall (*R*), and *mAP* (mean Average Precision)—to assess the training effectiveness of the disease detection model. Specifically, Precision represents the proportion of actual disease samples among those predicted as positive. It can be calculated using the following formula:

(12) $$P=\frac{TP}{TP+FP}$$

where *TP* denotes the number of samples where both the disease category and the bounding box are correctly predicted, and *FP* denotes the number of samples where either the disease category or the bounding box is correctly predicted. In the prediction results, a higher precision value indicates a lower rate of false positives in the disease detection model.

The recall represents the proportion of correctly predicted disease samples out of all annotated disease samples. It can be calculated using the following formula:

(13) $$R=\frac{TP}{TP+FN}$$

where *FN* denotes the number of samples where the disease category is incorrectly predicted, and the bounding box for the disease region is inaccurately identified. A higher recall value indicates a lower rate of missed detection by the disease detection model.

The *mAP* refers to the mean of average Precision (*AP*), where *AP* is the area under the Precision-Recall (P-R) curve. *mAP* can be calculated using the following formula:

(14) $$mAP=\frac{\sum_{i=1}^{N}\int_{0}^{1}P(R)dR}{N}\times 100\%$$

where *N* represents the number of citrus disease categories, with *N* = 6 in this study. In the prediction results, a higher *mAP* value indicates better performance in both classification and regression tasks across all disease categories, reflecting superior overall model performance.

This study also utilizes the TIDE metrics to provide a more comprehensive evaluation of the YOLOv8n-DE disease detection model’s performance [34]. The TIDE metrics used in this study include Classification Error (*Cls*), Localization Error (*Loc*), Both Cls and Loc Error (*Both*), Duplicate Detection Error (*Dupe*), Background Error (*Bkg*), and Missed Ground Truth Error (*Miss*). These metrics allow for separate calculations of classification, regression, false positives, and missed detections in the disease detection results, which improves the model’s interpretability.

Specifically, *Cls* represents the number of samples where the disease category is incorrectly predicted when *IoU_max_* ≥ 0.5, where *IoU_max_* refers to the bounding box with the highest Intersection over Union (*IoU*) with the disease target’s bounding box. *IoU* calculates the ratio of the intersection area to the union area of two bounding boxes. A lower *Cls* value indicates fewer classification errors by the disease detection model in the prediction results.

*Loc* represents the number of samples where the disease location is incorrectly predicted when 0.1 ≤ *IoU_max_* ≤ 0.5. In the prediction results, a lower *Loc* value indicates fewer errors in the localization of disease regions.

*Both* refer to the number of samples where the disease classification and location are incorrect when 0.1 ≤ *IoU_max_* ≤ 0.5. A lower *Both* value indicates fewer errors in the classification and localization of disease regions.

*Dupe* represents the number of samples where the target is detected multiple times when *IoU_max_* ≥ 0.5. A lower *Dupe* value indicates greater stability in the disease detection results.

*Bkg* represents the number of background regions mistakenly classified as targets in disease images. A lower *Bkg* value indicates better performance of the disease detection model.

*Miss* indicates the presence of disease targets in the image that the model failed to detect. A lower *Miss* value signifies the better performance of the disease detection model.

## 4. Experiments

### 4.1. Algorithm Ablation Experiments

**Figure 10:**
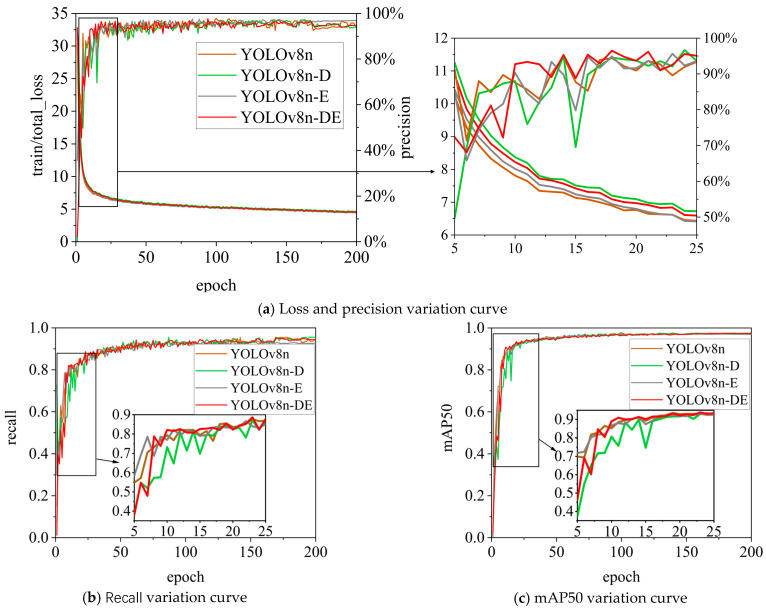
Variation curves of the YOLOv8n ablation experiment.

This section presents ablation experiments to analyze and validate the proposed YOLOv8n-DE citrus disease detection model. Ablation experiments were conducted comparing the YOLOv8n-DE disease detection model with the baseline YOLOv8 model. Figure 10 shows the variation of different metrics during the training process. YOLOv8n serves as the baseline model for the ablation experiments. YOLOv8n-D is the model obtained by replacing the C2f module in YOLOv8n with the DR module. YOLOv8n-E is the model obtained by replacing the detection head (Detect) in YOLOv8n with the Detect_Shared module. YOLOv8n-DE is the fast citrus disease detection model proposed in this paper, integrating the DR module with the Detect_Shared module, and is based on feature enhancement via dilated reparameterization and shared parameter heads.

Overall, the YOLOv8n-DE model demonstrates better convergence during training in terms of loss and precision. Regarding recall, YOLOv8n-DE performs well, maintaining a relatively high level. In terms of mAP50, YOLOv8n-DE shows strong stability and optimization ability. The curves in Figure 10 suggest that YOLOv8n-DE has excellent overall performance in disease detection tasks, providing accurate and stable results.

We evaluate each model’s disease detection performance using the test set based on the differences in loss, precision, recall, and mAP50 curves observed during training. Table 3 presents the detection performance of the models on the test set images, and we discuss the models’ generalization capabilities further.

The YOLOv8n-DE disease detection model integrates the advantages of YOLOv8n-D’s large convolution kernels and extended receptive field, along with the shared parameter head design and sparse data handling benefits of YOLOv8n-E, achieving optimal overall performance. Compared to the baseline YOLOv8n model, the YOLOv8n-DE model reduces the model size by 41.94%, the parameter count by 48.17%, and computation by 59.26%, while improving detection precision by 3%, recall by 0.1%, and mAP50 by 0.7%. These results show that, while balancing the model size and detection accuracy, the YOLOv8n-DE model achieves 97.6% precision, 91.8% recall, and 97.3% mAP50 with a model size of just 3.6 MB, 1.56 × 10^6^ parameters, and 3.3 GFLOPs of computation. This lightweight design makes YOLOv8n-DE suitable for deployment on edge devices and widespread use in orchards.

### 4.2. Sensitivity Testing of the Model to Specific Disease Categories

In addition to assessing the overall performance of the YOLOv8n-DE disease detection model, this study further evaluates the model’s capability to detect individual disease categories [35]. First, data from six disease categories—anthracnose (denoted as a), canker (c), Huanglongbing (h), melanose (m), nutrition deficiency (n), and Podagricomela nigricollis (p)—were separately fed into both YOLOv8n and YOLOv8n-DE models for training. This resulted in distinct YOLOv8n (*x*) and YOLOv8n-DE (*x*) models (where *x* represents the respective disease categories a, c, h, m, n, and p), producing a total of 12 single-class disease detection models. Subsequently, the models were trained on a mixed dataset containing all six disease categories, generating two multi-class disease detection models, allowing for analysis of how changes in the dataset influence the model’s performance in detecting various diseases.

**Figure 11:**
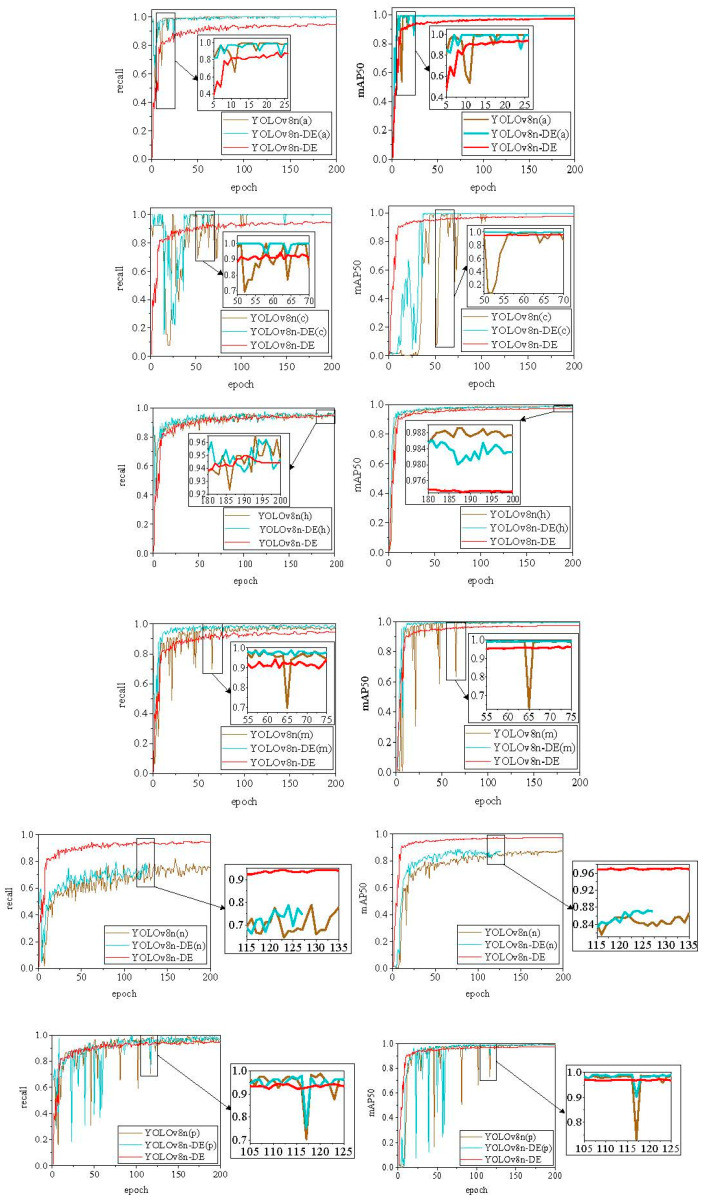
Comparison of the performance between single-class and multi-class citrus disease models. YOLOv8n (a) and YOLOv8n-DE (a) are models trained solely on the a-class disease. Similarly, YOLOv8n (c, h, m, n, p) and YOLOv8n-DE (c, h, m, n, p) are models trained on the c, h, m, n, and p disease classes, respectively. YOLOv8n and YOLOv8n-DE represent models trained on multiple disease classes.

The comparison between the single-class and multi-class disease detection models is shown in Figure 11. Experimental results reveal that the improved model, YOLOv8n-DE (*x*), exhibits slightly higher recall and mAP50 changes compared to the YOLOv8n (*x*) in the single-class models. Notably, in disease categories c, m, n, and p models, YOLOv8n-DE (*x*) converges faster than the baseline model, YOLOv8n (*x*). However, when comparing the YOLOv8n-DE models, it is evident that although YOLOv8n-DE (*x*) demonstrates higher recall and mAP50 changes in five of the disease categories (a, c, h, m, p) than the multi-class models, the training curves are less stable, showing occasional sharp drops. This instability indicates that overfitting may have occurred during the training of the single-class disease models. As shown in Figure 11, no overfitting was observed for the n disease category during the training of the single-class model, but its recall and mAP50 remained consistently low. In the case of YOLOv8n-DE (n), accuracy failed to improve, which triggered the early stopping mechanism after 128 epochs.

**Figure 12:**
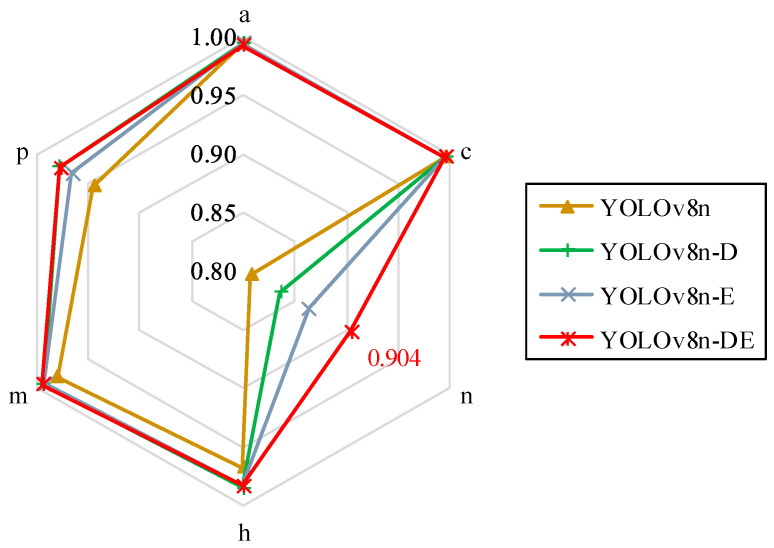
Radar chart of mAP50 for individual disease category tests.

The multi-class disease combination model, YOLOv8n-DE, demonstrated rapid convergence during the early stages of training (the first 20 epochs). In the subsequent training phases (post-20 epochs), the model exhibited no significant fluctuations, did not activate the early stopping mechanism, and the recall and mAP50 curves remained stable without any downward trend. These results suggest that YOLOv8n-DE offers better stability and a reduced risk of overfitting. Further testing was conducted on YOLOv8n-DE for detecting single-class diseases, and the results are presented in Figure 12 and Table 4.

Figure 12 shows that the disease detection model performed poorly for the nutrition deficiency (n) symptom while demonstrating high accuracy for other diseases. The YOLOv8n model exhibited the lowest detection accuracy across all categories, whereas the YOLOv8n-DE model consistently achieved the highest performance. A detailed analysis of the data in Table 4 reveals that YOLOv8n-DE achieved the highest mAP50 for both melanose (m) and nutrition deficiency (n), suggesting that the integration of the DR module and Detect_Shared module significantly improved the detection of these symptoms. For Podagricomela nigricollis (p), YOLOv8n-DE’s mAP50 is 97.7%, just 0.1% lower than the highest detection accuracy of 97.8% achieved by the YOLOv8n-D model. The YOLOv8n-DE model achieved an accuracy of 90.4% for n, a 9.7% improvement over the YOLOv8n model. The YOLOv8n-DE model showed the highest sensitivity to individual disease categories, performing well across all disease types tested.

Although class n diseases (708 samples) demonstrated substantially higher sample counts compared to class c (160 samples), they exhibited notably lower mAP50 scores (90.4% vs. other classes). Three primary factors contribute to class n’s reduced detection performance:

1. Symptom Ambiguity: Nutritional deficiency manifests through non-specific indicators like chromatic variation, growth retardation, and morphological abnormalities, contrasting with distinct visual markers in anthracnose or Huanglongbing.
2. Insufficient Sample Diversity: Despite relatively abundant samples (708), the dataset fails to comprehensively cover all nutritional deficiency variations, limiting model generalization.
3. Environmental Complexity: Orchard conditions involving variable illumination, heterogeneous leaf backgrounds, and co-occurring biotic/abiotic stressors particularly interfere with detecting subtle nutritional deficiency symptoms.

To systematically investigate dataset imbalance impacts, we conduct mean average precision analyses using YOLOv8n-DE under different data distributions (Table 5).

The experimental results in Table 5 indicate that sample imbalance had a notable impact on model performance. Removing the imbalanced “canker” category (NO.1) resulted in a modest decrease in mAP50 compared to the original dataset (NO.4), implying that even a skewed class can contribute useful information. Uniformly reducing each category to 160 images (NO.2) led to a substantial performance drop, highlighting the critical role of sufficient training data. In contrast, applying data augmentation techniques to oversample the underrepresented “canker” class (NO.3) significantly alleviated this degradation, although the performance still slightly lagged behind that of the original dataset. These results suggest that while augmentation can mitigate the negative effects of imbalance, maintaining the natural diversity and quantity of the original dataset ultimately yields the best model performance.

### 4.3. Evaluation of Model Fineness

Although the COCO metric is a widely adopted evaluation scheme in object detection, it needs to highlight the shortcomings of detection algorithms during testing effectively. This study comprehensively employs the TIDE metric to evaluate the model’s disease detection performance, offering a more in-depth analysis of the model’s weaknesses in certain aspects.

**Figure 13:**
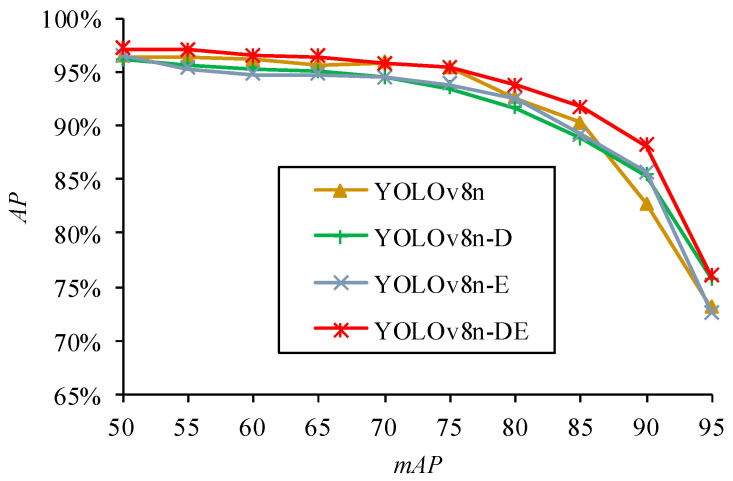
Segmented comparison of disease detection performance from mAP50 to mAP95.

Figure 13 presents a segmented comparison of the test results under varying precision requirements, with each segment corresponding to a different Intersection over Union (*IoU*) threshold. At *mAP* = 50, the AP values of all models exceed 95%, and the differences in mAP50 shown in Figure 13 are negligible. This observation aligns with the earlier statement that no significant differences were found in the loss curves, precision curves, and mAP50 curves during the model training process.

Table 6 compares the TIDE metrics for the disease detection models, which assess model misclassification and localization accuracy. YOLOv8n-DE demonstrates a 0.21 reduction in classification error (*Cls*) compared to YOLOv8n, representing a 42% improvement, indicating better disease classification performance. The localization error (*Loc*) decreases by 1.6, a 79.2% improvement, demonstrating that YOLOv8n-DE has a higher precision in target localization. The false positive (*FP*) metric decreases by 0.25, or 11.11%, improving classification and regression performance. The false negative (*FN*) metric decreases by 1.36, a 64.2% reduction, indicating that YOLOv8n-DE is particularly effective at detecting missed disease targets, providing a more comprehensive detection. The YOLOv8n-DE model excels in key TIDE metrics—*Cls*, *Loc*, *Dupe*, *Miss*, *FP*, and *FN*—demonstrating significant improvements over the other models.

Table 6 indicates that YOLOv8n-DE outperforms YOLOv8n, YOLOv8n-D, and YOLOv8n-E regarding classification accuracy, localization precision, and overall capability. These advantages make YOLOv8n-DE more accurate and reliable for disease detection in real-world applications.

Although the mAP50 metric shows comparable performance across the models, YOLOv8n-DE demonstrates a clear advantage at stricter *mAP* thresholds, exhibiting the most gradual decline in accuracy. This advantage is particularly evident in its ability to detect smaller, more complex disease regions under demanding conditions. TIDE metric analysis confirms that YOLOv8n-DE performs better than other models in various error metrics, showing a marked reduction in both localization and classification errors. These strengths contribute to YOLOv8n-DE’s superior accuracy and reliability in practical disease detection applications, particularly for high-precision scenarios.

### 4.4. Comparison of Detection Performance Among Different Algorithms

Due to the rapid updates and iterations of one-stage object detection algorithms, particularly those in the YOLO series, many researchers believe that the latest object detection algorithms offer higher efficiency, accuracy, and adaptability in detection tasks [36]. This study incorporates Programmable Gradient Information (PGI) and GELAN technologies from YOLOv9, as well as Non-Maximum Suppression (NMS) elimination techniques [37] from YOLOv10, into the YOLOv8 algorithm to assess further the effectiveness of different methods in disease detection tasks.

Table 7 compares the performance of the YOLOv8 algorithm when modified with the latest modules. The YOLOv8n-RepNCSPELAN model is a disease detection model that enhances the C2f module of the YOLOv8 model based on the GELAN concept from YOLOv9. The YOLOv8n-PGI model improves the YOLOv8 model by utilizing auxiliary training according to the PGI concept from YOLOv9. YOLOv8n-PGI_rep is a variant of the YOLOv8n-PGI model that removes the PGI model during the inference process, thus reducing inference costs. The YOLOv8n-nmsfree disease detection model incorporates a dual-label assignment strategy based on the NMS-free concept from YOLOv10 to improve the YOLOv8 algorithm.

As shown in Table 7, the disease detection models that integrate the latest advanced techniques, such as YOLOv8n-RepNCSPELAN, underperform in five key areas compared to the YOLOv8n-DE model proposed in this study: model size, parameters, computation, mAP50, and AP for the challenging class n (mAP50_n). Although the latest object detection algorithms, with their advanced techniques, demonstrate notable advantages on general datasets (e.g., COCO), they exhibit low efficiency, accuracy, and adaptability in citrus disease detection tasks.

**Figure 14:**
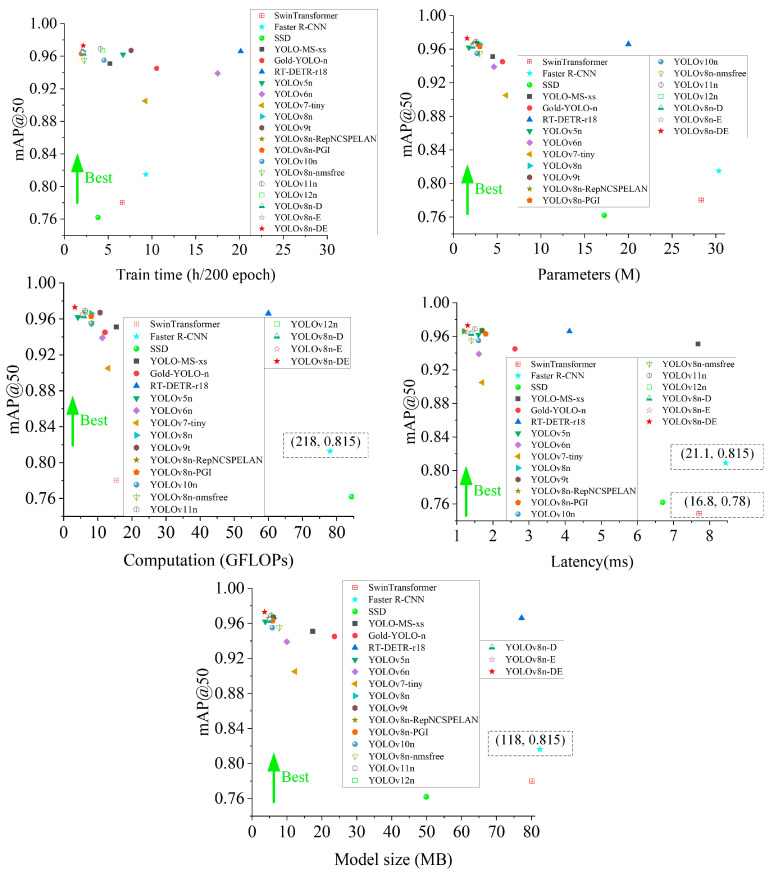
Variation in citrus disease mAP50 metrics across five dimensions.

**Figure 15:**
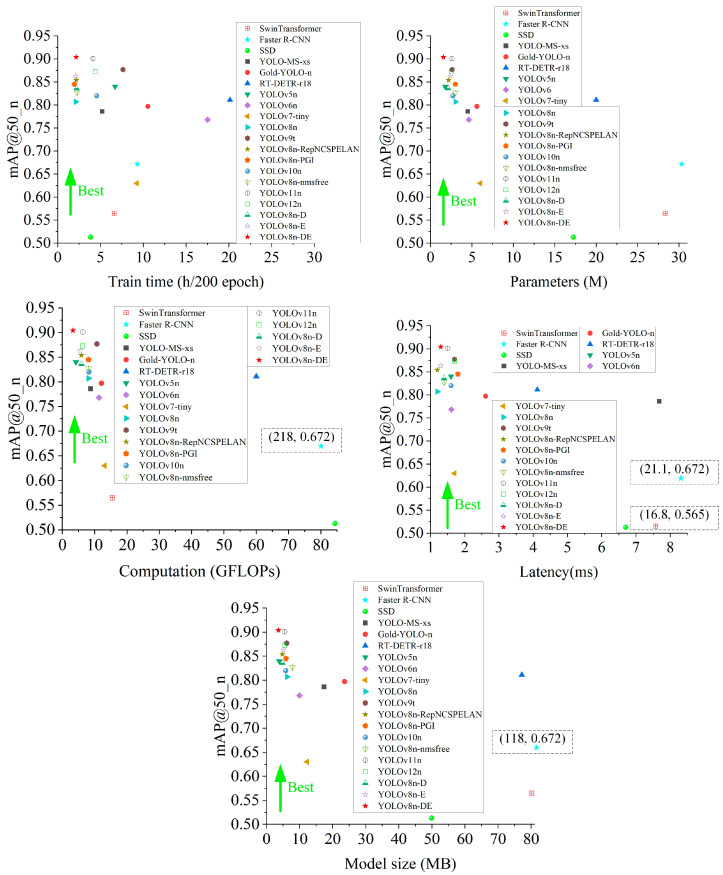
Variation in citrus disease mAP50 metrics for class n across five dimensions.

This study compares various algorithms for disease detection based on five dimensions: model training time, parameters, computation, latency, and model size. It analyzes the variations in overall mAP50 and the mAP50 for difficult-to-detect categories across different methods. The algorithms compared include: SwinTransformer [38], Faster R-CNN [39], SSD [40], YOLO-MS-xs [41], Gold-YOLO-n [42], RT-DETR-r18 [43] (transformer architecture), YOLOv5n [44], YOLOv6n [45], YOLOv7-tiny [46], YOLOv8n [20], YOLOv9t [21], YOLOv8n-RepNCSPELAN, YOLOv8n-PGI, YOLOv10n [37], YOLOv8n-nmsfree, YOLOv11 [47], YOLOv12n [48], YOLOv8n-D, YOLOv8n-E, and YOLOv8n-DE. The detection performance is illustrated in Figure 14 and Figure 15.

As shown in Figure 14 and Figure 15, under similar performance conditions, the YOLOv8n-DE model proposed in this paper achieves training times that are 3.06/4.3/2.1/3.53/4.88/9.31/8.09 times faster than SwinTransformer/Faster R-CNN/YOLOv10n/YOLOv9t/Gold-YOLO-n/RT-DETR-r18/YOLOv6n, respectively. YOLOv8n-DE demonstrates highly efficient utilization of parameters and computation, making it suitable for lightweight deployment. With a 3.86-fold reduction in parameters and a 3.97-fold reduction in computation, YOLOv8n-DE improves mAP50 by 6.8% and mAP50_n by 27.4% compared to YOLOv7-tiny. Additionally, with a 1.73-fold reduction in parameters and a 2.48-fold reduction in computation, YOLOv8n-DE improves mAP50 by 1.8% and mAP50_n by 8.4% compared to YOLOv10n. Compared to YOLO-MS-xs, YOLOv8n-DE reduces latency by 83.07% at the same performance level, and compared to YOLOv9t, it reduces latency by 23.53% under similar conditions. Regarding model size, YOLOv8n-DE is 95.34% smaller than RT-DETR-r18 and 40.98% smaller than YOLOv9t. YOLOv8n-DE achieves 0.4% and 0.6% mAP50 improvements over YOLOv11n and YOLOv12n, respectively, in general disease detection, with more significant 0.3% and 3.1% gains specifically for class n detection. Among all the algorithms compared, YOLOv8n-DE achieves the highest mAP50 across all disease detection tasks, reaching 97.3%. Furthermore, YOLOv8n-DE also achieves the highest mAP50 for class n diseases, with a value of 90.4%.

The YOLOv8n-DE model demonstrates significant advantages in disease detection tasks, offering lower latency, a smaller model size, and higher accuracy, all while reducing training costs, parameters, and computation. The YOLOv8n-DE disease detection model meets real-time and lightweight deployment requirements, showing exceptional practicality, efficiency, and precision in citrus disease detection.

### 4.5. Comparison of Lightweight Performance

**Figure 16:**
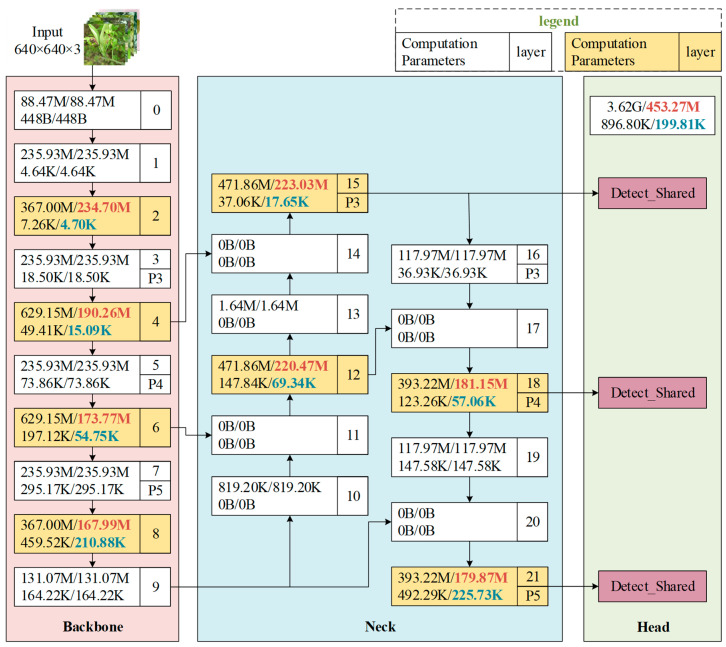
Reduction in computation and parameters of the YOLOv8n-DE model compared to the YOLOv8n model.

Using a test scale of 640 × 640, we compared the computational cost and parameter count of the YOLOv8n-DE model with those of the YOLOv8n model. The results are shown in Figure 16, where the module names in Figure 16 correspond to those in Figure 2. In the backbone network, both computation and parameters were reduced to four layers. The most significant reduction in computation occurred at layer 4, where it decreased from 629.15 M to 173.77 M, a reduction of 72.38%. The highest parameter reduction occurred at layer 6, where they dropped from 197.12 K to 54.75 K, a reduction of 72.23%. In the neck network, the most substantial reduction in computation was observed at layer 21, where it decreased from 393.22 M to 179.87 M, a reduction of 54.26%, and the parameters in this layer decreased from 492.29 K to 225.73 K, a reduction of 54.15%. In the head section, the overall computation decreased from 3.62 G to 453.27 M, a reduction of 7.99 times, while the parameters dropped from 896.80 K to 199.81 K, a reduction of 77.72%. Overall, the YOLOv8n-DE model resulted in a substantial reduction in both computational cost and parameter count compared to the YOLOv8n model.

The lightweight design of deep learning models focuses on reducing the number of parameters and computational cost and also considers inference speed (*FPS*, f/s). A minimum frame rate of 24 FPS is required for real-time object detection tasks. We tested the inference speeds of various models, and the experimental results are shown in Table 8.

Analysis of Table 8, in conjunction with Table 3 and Table 7 and Figure 14, reveals that while the FPS of YOLOv8n-DE is 781.44, slightly lower than YOLOv8n’s 796.13, YOLOv8n-DE has only 1.56 M parameters, nearly 50% fewer than YOLOv8n’s 3 M. The computational cost of YOLOv8n-DE (3.3 GFLOPs) is approximately 60% lower than YOLOv8n’s 8.1 GFLOPs. Regarding latency, YOLOv8n-DE and YOLOv8n are nearly identical, maintaining around 1.3 ms. This indicates that YOLOv8n-DE significantly reduces computational cost and parameter count while maintaining nearly the same inference speed as YOLOv8n. Furthermore, when compared to other models such as SwinTransformer, Faster R-CNN, YOLO-MS-xs, Gold-YOLO-n, and RT-DETR-r18, YOLOv8n-DE demonstrates particularly outstanding performance in both inference speed and efficiency. YOLOv8n-DE demonstrates 11.64× and 17.36× faster FPS compared to SwinTransformer and Faster R-CNN implementations.

In summary, the YOLOv8n-DE model significantly reduces model complexity and computational costs while maintaining excellent inference speed, greatly decreasing the hardware requirements. The YOLOv8n-DE model is highly efficient in resource utilization, enhancing its real-time processing capability for disease detection tasks and improving its lightweight performance in practical applications. YOLOv8n-DE maintains excellent inference speed and high detection accuracy by optimizing computational and storage demands, demonstrating strong potential for practical use.

## 5. Visualization Analysis

This study presents a visualization analysis of the YOLOv8n-DE disease detection model from three perspectives to enhance its interpretability. Specifically, the analysis includes (1) heatmap analysis to evaluate the model’s attention areas, (2) a comparison of the model’s inference results for various diseases, and (3) statistical analysis of detection counts in the test set, along with visualizations of correct detections, missed detections, and false detections in images with the highest error rates.

### 5.1. Heatmap Visualization

**Figure 17:**
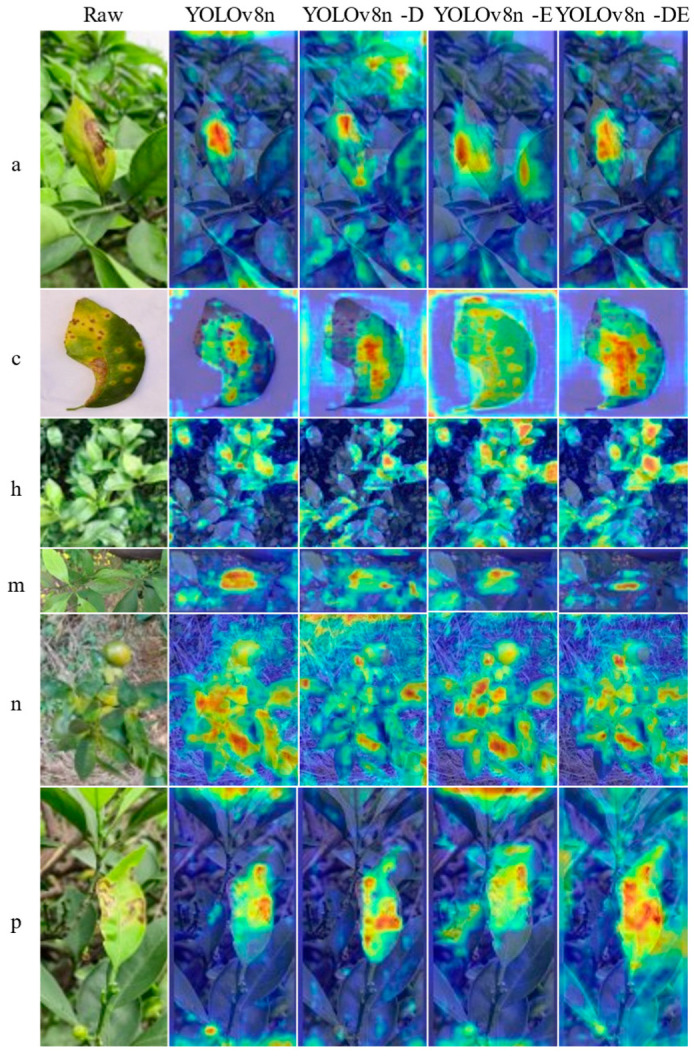
GradCAM visualization results.

Heatmap analysis can reveal the model’s focus on disease-related features, allowing us to assess whether the model has learned to attend to regions containing disease-specific characteristics [49]. Class Activation Mapping (CAM) techniques are commonly used in heatmap analysis. These methods include GradCAM, XGradCAM, EigenCAM, HiResCAM, LayerCAM, RandomCAM, and EigenGradCAM. In this study, we evaluated the performance of these seven CAM methods in disease detection models YOLOv8n, YOLOv8n-D, YOLOv8n-E, and YOLOv8n-DE. Ultimately, the GradCAM (Gradient-weighted Class Activation Mapping) method was selected for the disease visualization analysis. GradCAM visualization, an explainable AI technique for interpreting CNN predictions, operates through three stages: (1) Forward pass computation of image scores; (2) Gradient backward propagation and weighted aggregation for heatmap generation; and (3) ReLU-based heatmap normalization and overlay on test images. The GradCAM results are shown in Figure 17.

In the visualization results for a-Class diseases, YOLOv8n-DE effectively focuses on the diseased leaves, while YOLOv8n-D and YOLOv8n-E tend to over-focus on other background areas. For c-Class diseases, YOLOv8n and YOLOv8n-D show inadequate attention to the lesion areas, whereas YOLOv8n-E refines its focus on the lesion spots. In contrast, YOLOv8n-DE provides more concentrated attention to the diseased leaf areas. For h-Class diseases, YOLOv8n and YOLOv8n-E excessively focus on regions outside the diseased leaves, while YOLOv8n-D shows insufficient focus on the diseased leaves. In the visualization results for m-Class diseases, YOLOv8n over-focuses on non-diseased regions, and YOLOv8n-D and YOLOv8n-E show insufficient attention to the diseased leaves. YOLOv8n-DE, however, concentrates effectively on the diseased leaf areas. For n-Class diseases, YOLOv8n and YOLOv8n-E misidentify citrus fruits with similar colors as diseases, and YOLOv8n-D fails to focus on the diseased leaves. For p-Class diseases, YOLOv8n-DE appropriately directs attention to the diseased leaves, emphasizing the lesion areas.

Overall, the visualization results of the YOLOv8n-DE model, as proposed in this paper, demonstrate more focused attention, which allows it to capture the key features of various disease categories more accurately. The GradCAM visualization method enhances the model’s interpretability, showing that YOLOv8n-DE is better at highlighting lesion features, thereby achieving superior disease detection performance.

### 5.2. Model Recognition Performance Display

**Figure 18:**
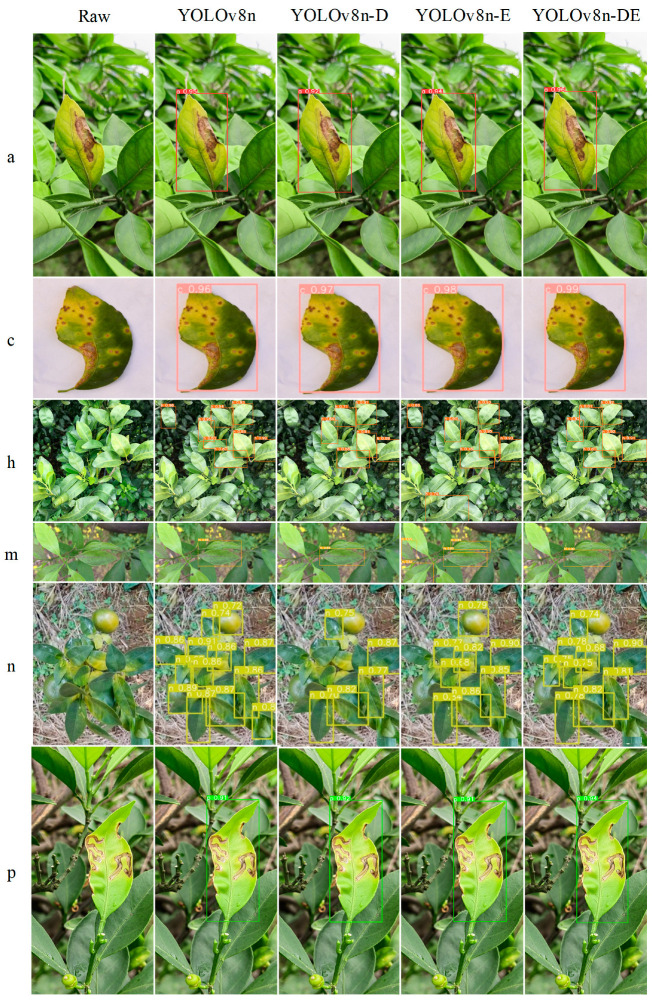
Disease recognition effect of different detection models.

The inference results of different object detection models on various diseases are presented in Figure 18. For detecting h-Class diseases, both YOLOv8n and YOLOv8n-E models displayed two false detections in different regions. In the m-Class disease detection, the YOLOv8n-E model showed three false detections. For n-Class diseases, both YOLOv8n and YOLOv8n-E models struggled to recognize shape features, misidentifying citrus fruits with similar colors as diseases. The YOLOv8n-D model missed nearly half of the diseased leaves. Based on the comparative experiments and disease recognition results discussed earlier, it is evident that the YOLOv8n-DE model, despite significant reductions in parameters and computation, maintains high accuracy in detecting various disease categories without any false or missed detections. The YOLOv8n-DE model consistently yields the highest confidence values across all disease categories.

### 5.3. Testing Results

Testing was conducted on the test set images with an Intersection over Union (*IoU*) threshold of 0.45. The detection results of the models are summarized in Table 9. Regarding false detections, the proposed YOLOv8n-DE model outperformed the other models. It reduced the number of false detections by 697 compared to the YOLOv8n model, by 513 compared to YOLOv8n-D (which incorporates only the DR module), and by 337 compared to YOLOv8n-E (which incorporates only the Detect_Shared module). These results demonstrate that the YOLOv8n-DE model is more effective at reducing false detection rates, which helps minimize erroneous identifications in practical applications, thereby improving the overall accuracy and reliability of the model.

**Figure 19:**
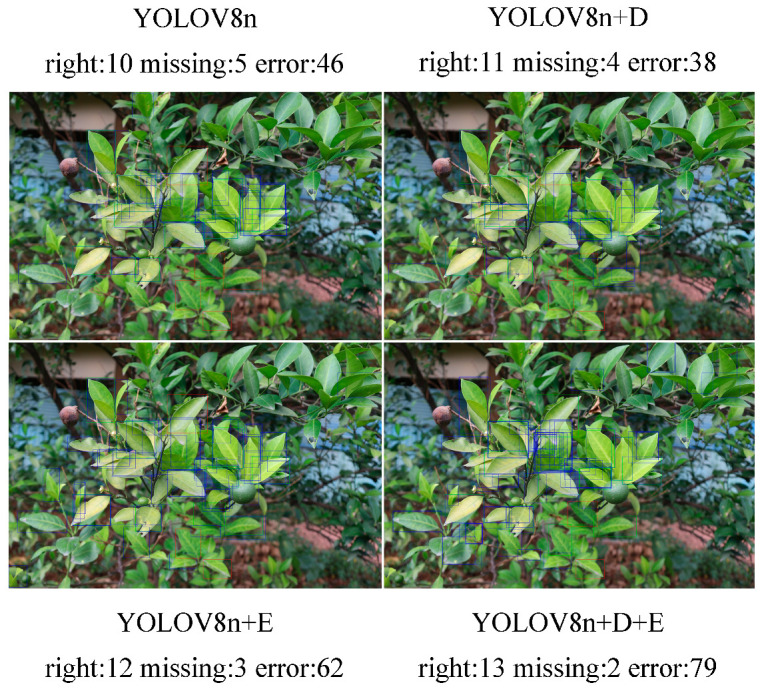
Visualization of the challenging n-Class disease detection image.

Figure 19 illustrates the visualization of the image with the highest detection error among all test images. This image represents the most difficult-to-detect n-Class disease, characterized by a complex background and indistinct disease features. Despite all models exhibiting missed detections on this challenging image, the YOLOv8n-DE model achieved the highest number of correct detections (YOLOv8n-DE: 13, YOLOv8n: 10, YOLOv8n-D: 11, YOLOv8n-E: 12) and the fewest missed detections (YOLOv8n-DE: 2, YOLOv8n: 5, YOLOv8n-D: 4, YOLOv8n-E: 3). While YOLOv8n-DE produced a relatively high number of false positives (79), its overall performance in terms of correct detections and missed detections was the best. These results indicate that YOLOv8n-DE performs better in handling complex backgrounds and challenging disease regions, reflecting stronger detection capability and robustness.

Despite superior detection performance in complex backgrounds (Figure 19), YOLOv8n-DE still exhibits occasional misclassifications and missed detections. Primary contributing factors include: (1) Background interference obscuring subtle disease features; (2) inherent symptom indistinctness; and (3) limited model generalization for extreme/atypical cases. As analyzed in Section 4.2, environmental complexity and feature ambiguity are particularly pertinent challenges in class n identification. Future enhancements could integrate multispectral data (visible, infrared, depth imagery) to improve robustness.

## 6. Discussion

The proposed YOLOv8n-DE model demonstrates a remarkable balance between lightweight design and detection accuracy in citrus disease detection tasks. YOLOv8n-DE achieves a model size of 3.6 MB and 1.56 M parameters, significantly outperforming recent works. Comparative analysis reveals that YOLOv8n-CDDA [50], a lightweight citrus disease detector (2.0 MB parameters, 45 FPS), achieves 88.36% mAP50 versus our 97.3%. Similarly, ref. [33] proposed a self-attention YOLOv8 variant for citrus diseases, achieving 92.5% mAP50, but with higher computational costs (6.5 GFLOPs vs. 3.3 GFLOPs in our model). For Citrus Greening Disease detection, model [51] requires 18.71 M parameters, potentially hindering edge deployment. This highlights YOLOv8n-DE’s superior parameter efficiency and computational frugality.

The proposed model achieves state-of-the-art accuracy for challenging categories like nutrition deficiency (90.4% mAP50), surpassing [52] who reported 86.7% mAP50 using YOLOv8-GABNet on similar tasks. Compared to baseline YOLOv8, YOLOv8-DE reduces classification and localization errors by 42% and 79.2%, respectively.

With a 781.44 FPS inference speed, our solution significantly outperforms YOLO-EAF [53] (94.4 FPS). The integration of dilated reparameterization and shared parameter heads enables YOLOv8n-DE to adapt to multi-scale disease features while minimizing redundancy. Notably, our model maintains robust generalization in complex orchard environments, addressing limitations identified in [54]’s PlantVillage-optimized approach (98% accuracy but poor field adaptability). Our model’s lightweight design (41.94% smaller than YOLOv8) ensures compatibility with edge devices, a critical advantage for orchard deployment.

Through systematic improvements in data acquisition, feature extraction (DR module), and detection head design (shared parameters), YOLOv8n-DE achieves customized optimization for citrus disease detection. Moreover, the training time of YOLOv8n-DE is 1.03, 0.91, 2.1, and 3.53 times faster than that of YOLOv12n, YOLOv11n, YOLOv10n, and YOLOv9t, respectively, indicating that the model has lower hardware requirements. However, this study has limitations, particularly the potential for identification errors when the model is applied in highly complex environmental settings. Future work could investigate integrating more diverse environmental data and multimodal information to enhance the model’s robustness. Additionally, optimizing the model’s computational efficiency and adaptability remains an important avenue for further research.

## 7. Conclusions

The proposed architectural modifications enhance detection accuracy and speed while reducing computational complexity, demonstrating practical viability for precision agriculture applications. This paper proposes YOLOv8n-DE with two key improvements: the DR module for feature enhancement, and Detect_Shared for parameter efficiency. The DR module improves disease feature extraction and expands the receptive field by combining dilated convolutions and reparameterization. Detect_Shared reduces parameters and computational complexity while maintaining high efficiency in classification and regression tasks using partial convolutions and channel fusion.

Compared with other models, YOLOv8n-DE shows a 0.4% increase in mAP50 over YOLOv11n and a 0.3% improvement in mAP50 for n-class disease detection. Similarly, the mAP50 value of YOLOv8n-DE is not significantly higher than that of YOLOv12n. However, YOLOv8n-DE outperforms these models in terms of detection speed, number of parameters, and computational load. Compared to the original YOLOv8, YOLOv8n-DE reduces parameters by 48.17%, computation by 59.26%, and model size by 41.94%, while decreasing classification error by 42%, regression error by 79.2%, FP by 11.11%, and FN by 64.2%. It achieves 97.6% precision, 91.8% recall, 97.3% mAP, and 90.4% mAP in the recognition of challenging diseases. The model maintains a high inference speed of 781.44 FPS.

## Figures and Tables

**Table 1:** Data distribution.

Disease Name	Label Name	Subset Quantity	Total Number of Images
Anthracnose	a	train: 996	1263
		val: 134	
		test: 133	
Canker	c	train: 134	160
		val: 13	
		test: 13	
Huanglongbing	h	train: 1138	1416
		val: 139	
		test: 139	
Melanose	m	train: 1419	1770
		val: 176	
		test: 175	
Nutrition Deficiency	n	train: 575	708
		val: 67	
		test: 66	
Podagricomela Nigricollis	p	train: 578	733
		val: 78	
		test: 77	

**Table 2:** Parameter definitions for the YOLOv8n-DE algorithm used in citrus disease detection.

Environmental Parameter	Value	Train-Parameter	Value
CPU	Intel (R) Xeon (R) Platinum 8375c CPU @ 2.90GHz	epochs	200
GPU	NVIDIA RTX A6000	batch_size	32
RAM	503 GB	workers	8
python	3.8.18	imgsz	640
opencv	4.8.0	optimizer	SGD
torch	1.12.0	lr0; lrf	0.01; 0.01
Framework	Pytorch (version 1.12.0)	weight_decay	0.0005
Cudnn	cudn11.3	momentum	0.937

**Table 3:** Ablation experiment results.

Model	Model Size (MB)	Parameters (M)	Computation (GFLOPs)	Precision (%)	Recall (%)	mAP50 (%)
YOLOv8n	6.2	3.01	8.1	94.6	91.7	96.6
YOLOv8n-D	4.8	2.15	5.9	92.8	91.8	96.3
YOLOv8n-E	5.1	2.42	5.5	94.8	92.1	96.5
YOLOv8n-DE	3.6	1.56	3.3	97.6	91.8	97.3

**Table 4:** Comparison of mAP50 for individual disease category tests.

Model	a (%)	c (%)	h (%)	m (%)	n (%)	p (%)
YOLOv8n	99.4	99.5	96.8	98.0	80.7	94.5
YOLOv8n-D	99.4	99.5	98.5	99.3	83.6	97.8
YOLOv8n-E	99.4	99.5	98.3	99.2	86.3	96.6
YOLOv8n-DE	99.3	99.5	98.3	99.4	90.4	97.7

**Table 5:** Performance of YOLOv8n-DE under different data distributions.

Datasets	Computation (GFLOPs)	mAP50 (%)
NO.1	3.3	95.8
NO.2	3.3	85.1
NO.3	3.3	96.9
NO.4	3.3	97.3

NO.1 represents the dataset excluding imbalanced class canker; NO.2 uses 160 samples per class; NO.3 applies data augmentation (oversampling, mosaic augmentation) to expand canker samples to 800; NO.4 denotes the original dataset.

**Table 6:** TIDE metrics comparison.

Model	*Cls*	*Loc*	*Both*	*Dupe*	*Bkg*	*Miss*	*FP*	*FN*
YOLOv8n	0.50	2.02	0.01	0.05	1.02	0.34	2.25	2.12
YOLOv8n-D	0.53	1.71	0.00	0.08	1.04	0.32	2.57	2.04
YOLOv8n-E	0.41	1.85	0.01	0.06	0.90	0.39	2.23	2.10
YOLOv8n-DE	0.29	0.42	0.00	0.03	0.92	0.00	2.00	0.76

**Table 7:** Performance of latest modules in disease detection tasks.

Model	Model Size (MB)	Parameters (M)	Computation (GFLOPs)	mAP50 (%)	mAP50_n (%)
YOLOv8n	6.2	3.01	8.1	96.6	80.7
YOLOv8n-RepNCSPELAN	4.8	2.19	5.9	96.6	85.4
YOLOv8n-PGI	10.4	4.26	11.3	96.3	84.5
YOLOv8n-PGI_rep	5.9	3.01	8.1	96.3	84.5
YOLOv8n-nmsfree	7.8	3.01	8.1	95.5	82.7
YOLOv8n-DE	3.6	1.56	3.3	97.3	90.4

**Table 8:** Comparison of inference speeds among different models.

Model	*FPS* (f/s)	Model	*FPS* (f/s)
SwinTransformer	67.14	YOLOv9t	752.94
Faster R-CNN	45.01	YOLOv8n-RepNCSPELAN	789.13
SSD	183.72	YOLOv8n-PGI	555.81
YOLO-MS-xs	276.25	YOLOv10n	776.32
Gold-YOLO-n	463.09	YOLOv8n-nmsfree	800.11
RT-DETR-r18	122.46	YOLOv11n	658.76
YOLOv5n	800.74	YOLOv12n	511.97
YOLOv6n	833.21	YOLOv8n-D	763.09
YOLOv7-tiny	516.21	YOLOv8n-E	802.44
YOLOv8n	796.13	YOLOv8n-DE	781.44

**Table 9:** Statistical results of disease detection.

Model	Right	Missing	Error
YOLOv8n	741	17	1705
YOLOv8n-D	741	17	1521
YOLOv8n-E	742	17	1345
YOLOv8n-DE	741	16	1008

## Data Availability

The PlantVillage, Citrus Plant Dataset, CCL’20, and AI challenger datasets are open-source datasets. Other data will be made available on request.
